# Ellipsoid Zone and External Limiting Membrane-Related Parameters on Spectral Domain-Optical Coherence Tomography and Their Relationships With Visual Prognosis After Successful Macular Hole Surgery

**DOI:** 10.3389/fmed.2021.779602

**Published:** 2021-11-10

**Authors:** Jiarui Yang, Huaqin Xia, Yushi Liu, Xinglin Wang, Hao Yuan, Qingyi Hou, Yimeng Ge, Yi Ding, Yuexin Wang, Changguan Wang, Xuemin Li

**Affiliations:** ^1^Department of Ophthalmology, Peking University Third Hospital, Beijing, China; ^2^Beijing Key Laboratory of Restoration of Damaged Ocular Nerve, Beijing, China

**Keywords:** idiopathic macular hole, optical coherence tomography (OCT), ellipsoid zone (EZ), external limiting membrane, visual prognosis

## Abstract

**Purpose:** To compare structural diameters of the ellipsoid zone (EZ) and external limiting membrane (ELM) bands on spectral domain-optical coherence tomography (SD-OCT) images between vision-improved (group A) and vision-unimproved (group B) patients, and investigate the connection between these parameters and visual prognosis.

**Materials and Methods:** Forty-five eyes of 43 patients with idiopathic full-thickness macular hole closed after vitrectomy were retrospectively reviewed. Best-corrected visual acuity (BCVA) and SD-OCT were conducted preoperatively and at 1 week, 1 month and 6 months postoperatively. Structural and functional parameters were then measured using ImageJ software.

**Results:** Among structural and functional parameters, the relative reflectivity of EZ and the ratio of continuous ELM and EZ in group A were significantly higher than in group B from the 1-month postoperative visit. At the 6-month follow-up, the diameter of EZ disruption in group A was significantly smaller than in group B, and the relative reflectivity of ELM/EZ was significantly higher than group B. At 6-months, BCVA was statistically significantly correlated with baseline BCVA, basal diameter (BD), macular hole index (MHI), and diameter of ELM/EZ disruption. Change in BCVA from baseline was found to be significantly correlated with axial length and diameter hole index (DHI).

**Conclusions:** Postoperative BCVA outcome was significantly correlated with integrity, thickness and reflectivity of the EZ band. Patients with smaller diameter of EZ disruption and higher reflectivity of EZ band tended to have better visual outcomes. Given that the EZ band reflects the recovery of mitochondria in photoreceptors, it is a promising parameter for their functional evaluation.

## Introduction

Idiopathic macular hole (MH), first described by Johnson and Gass in 1988 (1), is a full-thickness anatomical defect of the neural retina at the fovea that can lead to central vision loss. The standard treatment for MH is pars plana vitrectomy with internal limiting membrane (ILM) peeling and intravitreal gas tamponade, for which anatomic success rates of 85–100% have been reported (2–4). However, a sealed MH does not guarantee improvement in visual acuity despite the high surgical success rate, and studies have been conducted to identify the factors affecting the post-operative vision outcome of MH.

Optical coherence tomography (OCT) is a widely used non-invasive medical imaging technique that provides *in vivo* retinal images and is considered the current gold standard for MH diagnosis, staging, and monitoring (5–7). Recent developments in OCT have furthered the understanding of the healing process after MH closure and may provide insight into maximizing surgical success and predicting visual outcomes. Several OCT measurements including MH width, height, and volume, along with ellipsoid zone (EZ) alterations and external limiting membrane (ELM) features have been associated with successful MH closure and visual acuity improvement (8–13). However, limited data exist on the comprehensive evaluation of these OCT features after successful surgical repair, and it is not clear whether one single parameter or time point on OCT correlates best with visual outcome.

This study aimed to evaluate anatomical outcomes of MH surgery and their associations with visual function. We assessed OCT measurements of the EZ and ELM, including the newly developed EZ-related angle parameters of MH, in subjects with successfully closed idiopathic MH and identified factors affecting postoperative visual acuity outcomes.

## Materials and Methods

### Patients

This was a retrospective study approved by the Institutional Review Board of Peking University Third Hospital and conducted in accordance with the tenets of the Declaration of Helsinki. Subjects who met the following criteria were included in this study: diagnosed with stage 2, 3, or 4 MH between January 2018 and July 2020 with follow-up surgery; postoperative follow-up duration longer than 6 months and at least 3 visits; best-corrected visual acuity (BCVA) and spectral-domain OCT (SD-OCT) measured at each postoperative visit. We excluded patients with other retinal diseases such as age-related macular degeneration, diabetic retinopathy, retinal detachment, and epiretinal membrane, as well as patients whose MH was not successfully closed via primary surgery. Patients with newly developed cataract postoperatively were also excluded. Demographic information was obtained from medical records, and axial length was measured using IOL Master Biometry (Carl Zeiss Meditec, Jena, Germany).

All patients underwent 25-gauge pars plana vitrectomy, 0.25% indocyanine green was used to facilitate ILM peeling, and sterilized air tamponade was applied. All patients were instructed to maintain a prone position for at least 3 days. In cases with cataract, phacoemulsification was performed before vitrectomy. Slit lamp examinations were performed at every postoperative visit, and no obvious turbidity was found in the phakic eye. All patients provided written informed consent prior to surgery.

Patients were classified into two groups based on BCVA at the 6-month postoperative visit. Group A consisted of patients whose BCVA improved two lines or more on a standard logarithmic visual acuity chart, and those who improved less than 2 lines were included in group B. BCVA then was converted to logMAR units for statistical analysis, and ΔBCVA was defined as preoperative BCVA minus 6-month postoperative BCVA.

### OCT

OCT images were captured using SD-OCT in Autorescan mode (Spectralis; Heidelberg Engineering, Heidelberg, Germany) before the surgery and at 1 week, 1 month, and 6 months after surgery. Due to the delay in air absorption, qualified images were obtained in only 32 eyes (22 in group A and 10 in group B) at the 1-week postoperative visit. Minimum linear diameter (MLD) (14), basal diameter (BD) (14), height (H) of MH (15), diameter of ELM disruption, diameter of EZ disruption (16), maximum distance between EZ and retinal pigment epithelium (RPE), macular ILM-RPE distance, ELM reflectivity, EZ reflectivity, and RPE reflectivity were measured using ImageJ software (1.47v, Wayne Rasband, National Institutes of Health, Bethesda, MD, USA, http://imagej.nih.gov/ij) on the horizontal orientation of the OCT image. MLD was defined as the minimal extent of the hole (14). BD was defined as the diameter of the hole at the level of the RPE (14). H was defined as the vertical distance from the midpoint of the nerve fiber layer on both edges to the RPE (15). Diameter of ELM disruption was defined as the distance between the two edges at the ELM, as was the diameter of EZ (16). Other indexes were calculated using the above parameters: diameter hole index (DHI)=MLD/BD, MH index (MHI)=H/BD, and traction hole index (THI) = H/MLD (17). The absolute reflectivity of ELM, EZ and RPE were obtained using the “plot profile” function of ImageJ (18). This function drew the reflectivity graph from a straight line through the center of the fovea. As the outermost highly reflective band was thought to represent the RPE, we regarded the highest value as the reflectivity of the RPE, the high reflectivity value adjacent to the RPE band was assumed to represent the EZ band, and high reflectivity value adjacent to the EZ band was assumed to represent the ELM band. If the EZ or ELM band was disrupted at the fovea, the reflectivity at the corresponding position was recorded. Relative reflectivity was calculated according to the following formula: Relative reflectivity (arbitrary unit) = (reflectivity of EZ or ELM)/(reflectivity of RPE)x100 (18).

**Figure 1:**
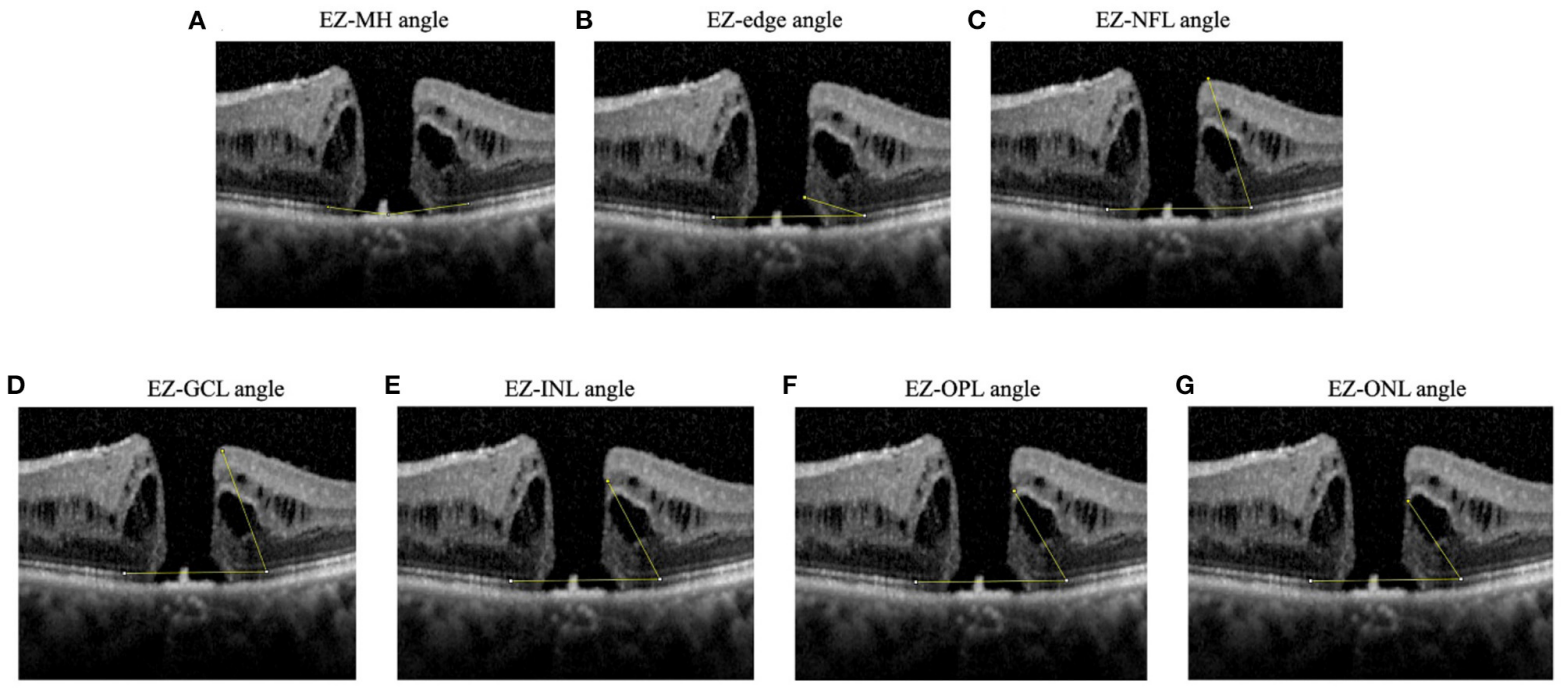
Diagrams showing angles measured. **(A)** EZ-MH angle: the angle whose vertex located at the center of the hole and the endpoints of both sides were located at the anterior border of the EZ band edge, **(B)** EZ-edge angle: the angle between a line connecting the upper edge of the EZ band and a line through the most protruding point of either edge of the MH, **(C)** EZ-NFL angle, **(D)** EZ-GCL angle, **(E)** EZ-INL angle, **(F)** EZ-OPL angle, **(G)** EZ-ONL angle. The EZ-NFL, EZ-GCL, EZ-INL, EZ-OPL, and EZ-ONL angles were each between a line connecting the two upper edges of the EZ band, and a line through the upper edge of the respective band. If measurements were different between left and right hole edge, the smaller angle was recorded. EZ, ellipsoid zone; GCL, ganglion cell layer; INL, inner nuclear layer; NFL, nerve fiber layer; ONL, outer nuclear layer; OPL, outer plexiform layer.

Additionally, we developed new EZ-related structural parameters including EZ-MH angle, EZ-edge angle, EZ-nerve fiber layer (EZ-NFL) angle, EZ-ganglion cell layer (EZ-GCL) angle, EZ-internal nuclear layer (EZ-INL) angle, EZ-outer plexiform layer (EZ-OPL) angle, and EZ-outer nuclear layer (EZ-ONL) angle. The EZ-MH angle referred to the angle whose vertex was located at the center of the hole, and the endpoints of both sides were located at the anterior border of the EZ band edge. The EZ-edge angle was between a line connecting the upper edge of the EZ band and a line through the most protruding point of either edge of the MH. The EZ-NFL, EZ-GCL, EZ-INL, EZ-OPL, and EZ-ONL angles were each between a line connecting the two upper edges of the EZ band, and a line through the upper edge of the respective band. We then take the average angle of two sides for analysis (Figure 1). All measurements were performed twice by the same clinician and the average of the two measurements were used for analysis.

### Statistical Analysis

All statistical analyses were performed using IBM SPSS for Mac version 26.0 (IBM Corp., Armonk, NY, USA). The repeatability was excellent for the measurement of structural parameters (intraclass correlation coefficient > 0.90 for all parameters). Parameters between the two groups before surgery and 1 week, 1 month, 6 months after surgery were compared using independent-sample *t-*tests if they showed homogeneity of variance on Levene's test, otherwise non-parametric analyses were used. Repeated-measures analysis of variance was used to compare BCVA, diameter of EZ/ELM disruption, and ELM/EZ relative reflectivity over time. Chi-square tests were used to compare whether the rate of closed ELM/EZ band in group A was higher than that in group B at the 1- and 6-month follow-up visits. Spearman's correlation analysis was used to test the correlation between each preoperative parameter and baseline BCVA, ΔBCVA, and BCVA at 6 months. *P* < 0.05 was considered statistically significant.

## Results

### Demographics and Baseline Data

Initially, 46 eyes were included in this study, of which 1 eye with MH recrudescence at the 6-month postoperative visit was excluded. Therefore, 45 eyes (19 OD, 26 OS) of 43 patients (12 male, 31 female) with a mean (± standard deviation) age of 64.33 ± 7.60 years were included in this study. Sixteen eyes were classified as stage 2 MH, nine eyes as stage 3, and twenty eyes as stage 4. The mean BCVA was 0.98 ± 0.33 logMAR; the mean axial length was 23.83 ± 1.36 mm; and the mean MLD, BD, and H were 578.46 ± 339.65 μm, 1319.17 ± 575.10 μm, and 439.87 ± 105.53 μm, respectively. The mean preoperative diameters of ELM and EZ disruption were 2049.94 ± 700.77 μm and 2379.87 ± 799.06 μm, respectively (Table 1).

**Table 1:** Demographic data^a^.

**Item**	**Value**
Number of eyes	45
Sex, M/F	12/31
Eye, OD/OS	19/26
Age, years	64.33 ± 7.60
Axial length, mm	23.83 ± 1.36
MH stage, *n* (%)	
2	16 (35.6%)
3	9 (20.0%)
4	20 (44.4%)
BCVA, Log MAR	0.98 ± 0.33
MLD, μm	578.46 ± 339.65
BD, μm	1319.17 ± 575.10
H, μm	439.87 ± 105.53
Preoperative diameter of ELM disruption, μm	2049.94 ± 700.77
Preoperative diameter of EZ disruption, μm	2379.87 ± 799.06

a *All values are mean±standard deviation unless otherwise indicated. BCVA, best-corrected visual acuity; BD, basal diameter; H, height; ELM, external limiting membrane; EZ, ellipsoid zone; MLD, minimal linear diameter*.

On a standard logarithmic visual acuity chart, BCVA improved two lines or more in 30 eyes (group A) and less than 2 lines in 15 eyes (group B). Demographic parameters such as age, axial length, and proportion of patients at each MH stage showed no significant differences between groups (*P* = 0.902, *P* = 0.729). Baseline BCVA was also similar in the two groups (*P* = 0.096). From the structural perspective, BD was significantly smaller in group A than group B (*P* = 0.031). Distance parameters MLD (*P* = 0.276), H (*P* = 0.277), diameter of ELM and EZ disruption (*P* = 0.149 and 0.710, respectively), maximum distance between EZ and RPE (*P* = 0.444), and fovea ILM-RPE distance (*P* = 0.621) showed no significant difference between groups. Indexes calculated based on the above parameters were also similar between groups (DHI, *P* = 0.886; THI, *P* = 0.148; FC, *P* = 0.886). All angle parameters were comparable in both groups (EZ-MH angle, *P* = 0.866; EZ-end angle, *P* = 0.084; EZ-NFL angle, *P* = 0.485; EZ-GCL angle, *P* = 0.337; EZ-INL angle, *P* = 0.146; EZ-OPL angle, *P* = 0.177; EZ-ONL angle, *P* = 0.219) (Table 2).

**Table 2:** Preoperative parameter comparisons between groups A and B^a^.

	**Group A**	**Group B**	***P* value**
Number	30	15	
Age, years	64.43 ± 7.99	64.13 ± 7.01	0.902
MH stage			
2	11	5	
3	5	4	0.729
4	14	6	
Axial length, mm	23.56 ± 1.23	24.38 ± 1.49	0.100
BCVA, Log MAR	0.93 ± 0.32	1.10 ± 0.34	0.096
MLD, μm	535.02 ± 335.16	659.13 ± 345.35	0.276
BD, μm	1181.49 ± 486.79	1584.69 ± 654.54	0.031*
H, μm	424.68 ± 85.28	469.17 ± 135.34	0.277
Diameter of ELM disruption, μm	1934.69 ± 712.57	2280.45 ± 641.05	0.149
Diameter of EZ disruption, μm	2345.52 ± 870.04	2448.56 ± 661.24	0.710
Maximum distance between EZ and RPE, μm	26.20 ± 8.60	28.89 ± 12.77	0.444
Fovea ILM-RPE distance, μm	51.51 ± 13.81	49.00 ± 16.07	0.621
DHI	0.43 ± 0.18	0.42 ± 0.18	0.886
MHI	0.42 ± 0.20	0.33 ± 0.13	0.148
THI	1.09 ± 0.71	0.91 ± 0.49	0.413
Angle			
EZ-MH	168.63 ± 4.59	168.35 ± 5.30	0.866
EZ-end	38.51 ± 9.57	44.61 ± 11.45	0.084
EZ-NFL	68.34 ± 12.67	71.31 ± 12.75	0.485
EZ-GCL	65.43 ± 12.23	69.30 ± 11.63	0.337
EZ-INL	58.38 ± 13.83	64.49 ± 9.71	0.146
EZ-OPL	54.15 ± 14.07	60.04 ± 10.73	0.177
EZ-ONL	50.40 ± 14.47	55.95 ± 11.43	0.219

a All values are mean±standard deviation unless otherwise indicated. BCVA, best-corrected visual acuity; BD, basal diameter; ELM, external limiting membrane; EZ, ellipsoid zone; GCL, ganglion cell layer; H, height; ILM, internal limiting membrane; INL, inner nuclear layer; MLD, minimal linear diameter; NFL, nerve fiber layer; ONL, outer nuclear layer; OPL, outer plexiform layer; RPE, retinal pigment epithelium. *P < 0.05.

### Functional and Anatomic Rehabilitation

**Figure 2:**
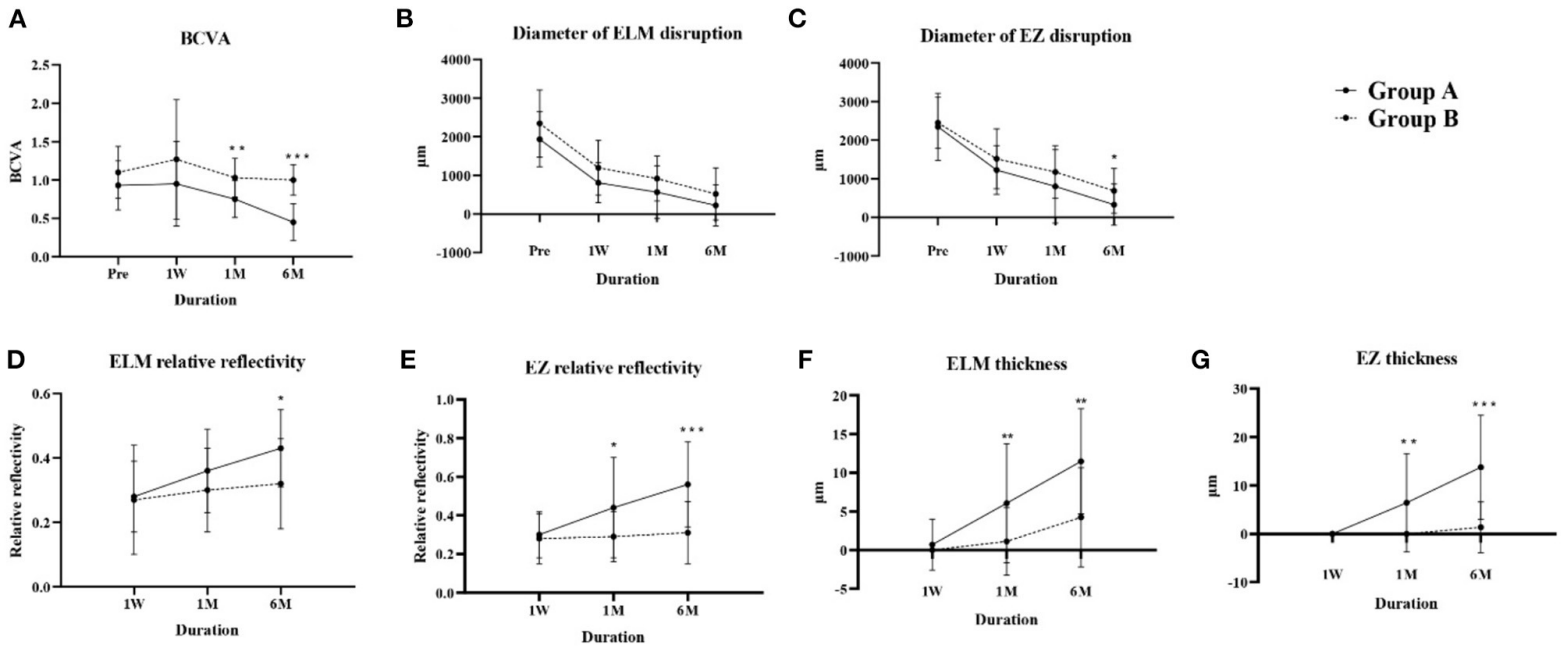
Changes in parameters over time in groups A and B. **(A)** BCVA, **(B)** diameter of ELM disruption, **(C)** diameter of EZ disruption, **(D)** ELM relative reflectivity, **(E)** EZ relative reflectivity, **(F)** ELM thickness, **(G)** EZ thickness. BCVA, best-corrected visual acuity; ELM, external limiting membrane; EZ, ellipsoid zone. *P < 0.05; **P < 0.01; ***P < 0.001.

BCVA changes over time were significantly different between groups (*P* < 0.001). BCVA in both groups first declined at 1 week compared with baseline, then improved at 1 and 6 months postoperatively. BCVA in group A improved from 0.93 ± 0.32 at baseline to 0.45 ± 0.24 at 6 months and in group B from 1.10 ± 0.34 to 1.00 ± 0.20, and BCVA differed between groups at 1 and 6 months postoperatively (*P* = 0.001 and *P* < 0.001 respectively) (Figure 2A).

The ELM and EZ disruption diameters reduced at each postoperative visit in both groups. Although the change over time, analyzed by repeated-measures analysis of variance, showed no significant difference between groups A and B (*P* = 0.961 and *P* = 0.706 respectively), Group A had less EZ disruption at 6 months postoperatively (*P* = 0.045) (Figures 2B,C).

Reflectivity is reportedly an essential parameter when evaluating the recovery of ELM and EZ bands (18). Both the absolute (Supplementary Figure 1) and relative reflectivity of ELM and EZ bands (Figures 2D,E) increased over time in both groups, and both the absolute and relative reflectivity of the EZ band in group A was higher than that in group B at 1 month (*P* = 0.024, *P* = 0.015) and 6 months (*P* < 0.001, *P* < 0.001) postoperatively, and for the ELM band at 6 months (*P* = 0.012, *P* = 0.015).

Foveal ILM-RPE thickness showed no significant difference at any time between the two groups (Supplementary Table 1), while group A showed greater ELM and EZ thickness compared with group B at the 1-month (*P* = 0.009, *P* = 0.001) and 6-month (*P* = 0.001, *P* < 0.001) postoperative visits (Figures 2F,G). Our results indicate that the thicknesses and reflectivity of EZ and ELM might better predict visual rehabilitation than integrity indicators.

### Factors Associated With Visual Rehabilitation

Due to unabsorbed intraocular gas in 13 eyes at the 1-week postoperative visit, a total of 32 OCT images were captured at this time point. In group A, ELM was restored in one (4.5%) eye at the 1-week postoperative visit, 12 (40%) eyes at 1 month, and 23 (76.7%) eyes at 6 months postoperatively. No eyes with continuous EZ bands were found in group A at 1-week; this band was intact in 9 (30%) eyes and 19 (63.3%) eyes at 1 and 6 months, respectively. In Group B, none of the eyes showed an intact ELM band at the 1-week postoperative visit, and the band was restored in 1 (6.7%) eye and 5 (33.3%) eyes at 1- and 6-months respectively. The EZ band was intact only in one (6.7%) eye at the 6-month postoperative visit (Table 3). At the 1-month visit, the ratio of intact ELM and EZ were both significantly higher than in group B (*P* = 0.034 and *P* = 0.020, respectively) and remained significantly higher at 6 months (*P* = 0.008 and *P* < 0.001, respectively).

**Table 3:** Cases of ELM/EZ restored in groups A and B over time.

		**Group A (intact/disrupted)**	**Group B (intact/disrupted)**	***P* value**
ELM	Postoperative 1 Week	1/21	0/10	1.000
	Postoperative 1 Month	12/18	1/14	0.034*
	Postoperative 6 Months	23/7	5/10	0.008*
EZ	Postoperative 1 Week	0/22	0/10	-
	Postoperative 1 Month	9/21	0/15	0.020*
	Postoperative 6 Months	19/11	1/14	<0.001*

ELM, external limiting membrane; EZ, ellipsoid zone. *P < 0.05.

In further exploration of the predictive value of baseline parameters for visual rehabilitation, correlation analysis showed a significant correlation between baseline BCVA and preoperative diameter of ELM disruption (*r* = 0.350, *P* = 0.029), MLD (*r* = 0.334, *P* = 0.035), DHI (*r* = 0.366, *P* = 0.019), MHI (*r* = −0.376, *P* = 0.015), and THI (*r* = −0.393, *P* = 0.012). BCVA at 6 months was significantly correlated with baseline BCVA (*r* = 0.615, *P* < 0.001), BD (*r* = 0.398, *P* = 0.010), MHI (*r* = −0.392, *P* = 0.011), preoperative diameter of ELM (*r* = 0.461, *P* = 0.003), and EZ disruption (*r* = 0.395, *P* = 0.013). ΔBCVA was significantly correlated with axial length (*r* = −0.424, *P* = 0.013) and DHI (*r* = 0.435, *P* = 0.004) (Table 4). Our results showed that BCVA outcome was strongly correlated with baseline BCVA and that no EZ-related parameter was predictive of BCVA improvement.

**Table 4:** Spearman's correlation analysis between preoperative factors and baseline BCVA, 6-month postoperative BCVA and ΔBCVA.

**Item**		**Baseline BCVA**	**6M BCVA**	**ΔBCVA**
Age	r	−0.228	−0.048	−0.143
	P	0.132	0.753	0.347
Baseline BCVA, Log MAR	r	-	0.615	0.250
	P	-	<0.001*	0.097
Axial length	r	−0.227	0.098	−0.424
	P	0.197	0.580	0.013*
Preoperative diameter of ELM disruption	r	0.350	0.461	−0.198
	P	0.029*	0.003*	0.227
Preoperative diameter of EZ disruption	r	0.211	0.395	−0.246
	P	0.197	0.013*	0.132
Macular ILM–RPE distance	r	−0.068	−0.028	−0.063
	P	0.691	0.871	0.709
MLD	r	0.334	0.273	0.048
	P	0.035*	0.298	0.768
BD	r	0.273	0.398	−0.200
	P	0.084	0.010*	0.209
H	r	−0.111	0.078	−0.207
	P	0.489	0.629	0.194
DHI	r	0.366	−0.007	0.435
	P	0.019*	0.965	0.004*
MHI	r	−0.376	−0.392	0.105
	P	0.015*	0.011*	0.515
THI	r	−0.393	−0.205	−0.182
	P	0.012*	0.204	0.262

BCVA, best-corrected visual acuity; 6M BCVA, 6-month best corrected visual acuity; BD, basal diameter; ELM, external limiting membrane; EZ, ellipsoid zone; H, height; ILM, internal limiting membrane; MLD minimal linear diameter; RPE, retinal pigment epithelium. ΔBCVA was defined as preoperative BCVA minus 6-month postoperative BCVA. *P < 0.05.

## Discussion

The role of EZ and ELM bands in the recovery of BCVA following MH repair has attracted much attention in recent years. Here we divided MH patients into two groups based on whether their BCVA improved at the 6-month postoperative visit and analyzed the pre- and postoperative parameters including the EZ and ELM bands to explore whether they could predict BCVA outcome. To our knowledge, this is the first study to explore differences of structural and functional parameters of EZ band between BCVA-improved patients and BCVA-unimproved patients. Our results showed that EZ and ELM recovery are strongly related to visual rehabilitation, suggesting that the EZ and ELM bands played important roles in visual recovery. Baseline BCVA correlated most strongly with BCVA outcome, and ΔBCVA correlated only with DHI and axial length.

Currently, pars plana vitrectomy with ILM peeling is the standard treatment for MH, but successful MH closure does not suggest the recovery of BCVA, and some studies have found BCVA recovery to be closely related to EZ band status (13, 19). In the present study, the parameters used to evaluate the EZ band included integrity, thickness, angle and reflectivity. For integrity of EZ band, Ooka et al. reported that EZ disruption at 1, 3, and 6-months postoperatively were closely correlated with BCVA (20). de Sisternes et al. showed that less EZ disruption indicated better BCVA outcome (21). Our study found that the ratio of closed EZ band in Group A was significantly higher than in Group B at 1- and 6-months postoperative visits, showing a positive correlation between EZ band recovery and BCVA, which is consistent with previous studies. In terms of thickness, Lee et al. found that BCVA was affected by inner retinal layer thickness, which was in turn significantly associated with EZ recovery (22). A study by Sevgi et al. showed that EZ-RPE thickness at 1-month follow-up in MH patients was significantly correlated with BCVA at 12 months postoperatively (23). We found that ELM and EZ in group A were significantly thicker than in group B at the 1- and 6-month postoperative visits, suggesting that recovery of ELM and EZ band thicknesses also indicate better BCVA outcome. As for angle, we initially hypothesized that the EZ-related angle reflected MH structural aspects and may be one of the predictive factors for BCVA recovery. However, although parameters including a range of EZ angles were larger in group B than in group A, the difference was not statistically significant. Chhablani et al. found no significant correlation between MH angle and final visual acuity (24), which correlated with our results. However, in some patients with high myopia, posterior scleral staphyloma may affect the measurement of these angles mentioned above. Of the 45 eyes involved in this study, only one eye had posterior scleral staphyloma, thus we did not separate this patient for analysis. In future research, it is necessary to explore the influence of the posterior pole scleral morphology on angle measurement. In terms of reflectivity, Tuan et al. found that the absolute and relative reflectivity of the EZ band gradually increased after MH surgery and were significantly correlated with improvement in BCVA (18). To exclude the influence of the image itself, we used RPE reflectivity as a reference to obtain the relative reflectivity for analysis. Relative reflectivity of EZ band in group A was higher than in group B from 1 month postoperatively, and the ELM showed a similar difference at the 6-month visit. These results suggest that relative reflectivity is related to BCVA and could be a promising parameter to describe the EZ function.

The ELM is the outermost hyperreflective band on SD-OCT and is thought to be composed of Müller cell terminal processes and microvilli (25). From a pathological perspective, it was hypothesized that Müller cells provide primary structural support for the fovea, acting like a plug to bind together photoreceptor cells (26). Müller cell dysfunction would increase susceptibility to MH formation, so Müller cell recovery is thought to be essential for retinal structure repair and later for visual rehabilitation. From a clinical perspective, Padron-Perez et al. demonstrated that ELM recovery after treatment was positively correlated with visual acuity recovery (27). Consistent with this, our results showed that the ELM closure rate was higher in group A than group B at 1 and 6 months postoperatively.

The EZ band is adjacent and anterior to ELM, the second outer hyperreflective band on SD-OCT, and its anatomical correlation remains contentious (28). It was previously believed to represent the junction between the inner and outer segments (IS/OS junction) of the photoreceptors (29). Recent research suggests that it is anatomically related to (and named for) the ellipsoid portion of the photoreceptor inner segment, a cellular compartment containing numerous mitochondria (28). Since mitochondria are the most light-scattering organelles, this explanation for a hyper reflective band seems reasonable (30). As a result, the recovery of the EZ band after MH surgery is thought to represent the enrichment of mitochondria generating cellular energy in the photoreceptors (25), and therefore EZ recovery may indicate improved photoreceptor function. In our study, the relative reflectivity of EZ in group A was significantly higher than that of group B from the 1-month visit and after and was connected to BCVA improvement. Therefore, we speculate that reflectivity may be a promising indicator of mitochondria recovery, which then extending to the recovery of photoreceptor function. More studies are needed to confirm correlation between EZ reflectivity and functional recovery of photoreceptors, and the measurement of reflectivity needs to be standardized.

The ELM and EZ bands are closely connected. Bottoni et al. reported that the ELM was the first structure to recover after MH closure (9). Lee and colleagues demonstrated that ELM and EZ recovery occurred 1.5 and 6.1 months after full-thickness MH, respectively, and all subjects with intact EZ showed intact ELM, indicating that ELM may be a prerequisite for EZ recovery (22). In our study, the rate of EZ recovery was lower than that of the ELM, and patients with an intact EZ also had an intact ELM, supporting the hypothesis that their recoveries are closely connected. We speculate that Müller cell recovery may be helpful to enhance mitochondria numbers in photoreceptors. However, we only recorded structural changes in the first 6 months after surgery, and it is possible that once ELM integrity is restored, EZ recovery might be observed over long-term follow-up.

Although EZ recovery was found connected with visual rehabilitation in our study, no preoperative EZ-related parameter was significantly different between the two groups, and BD appeared to be more valuable for visual prognosis. A study by Sevgi et al. showed that baseline MH width and volume were negatively correlated with postoperative BCVA (23). Our research revealed that mean MLD, mean BD, and mean diameter of ELM and EZ disruption in group A were smaller than those in group B, but only BD was statistically significant. Spearman's correlation analysis showed that 6-month BCVA was mainly determined by the baseline BCVA, BD, MHI, and diameter of ELM/EZ disruption, among which baseline BCVA showed the strongest significance, indicating that patients with better baseline BCVA tend to have better BCVA outcomes. ΔBCVA was significantly correlated with axial length and DHI at baseline. Lee et al. found that long axial length may impair EZ recovery, resulting in poorer functional visual outcomes (22), which is consistent with our finding of a negative correlation between axial length and ΔBCVA. As no single EZ-related preoperative parameter differed significantly between groups A and B, a larger sample size and measurement of other OCT parameters related to the EZ band are needed in future research investigating the close relationship between the EZ band and photoreceptor function. The effects of different treatment strategies on the EZ band also need to be clarified.

Our study had several limitations. First, the number of eyes included was not large, and the 6-month follow-up time was relatively short. Second, our data were collected in clinical practice, so there was a lack of uniform protocols to ensure consistency for patients at different clinical visits. Third, we only evaluated BCVA to assess visual function, and adding more parameters such as visual field tests would make the results more comprehensive. Last, we only measured the horizontal direction of OCT image, and future study should focus on all directions of OCT images to comprehensively analyze the structural parameters.

In conclusion, this study demonstrated significant correlations between EZ band integrity, thickness, and reflectivity and postoperative BCVA outcome. Patients with smaller diameter of EZ disruption and higher EZ band reflectivity tend to have better BCVA. Moreover, given that the EZ band reflects mitochondria recovery in photoreceptors, related parameters could be useful for evaluation of photoreceptor function after MH treatment.

## Data Availability Statement

The raw data supporting the conclusions of this article will be made available by the authors, without undue reservation.

## Ethics Statement

The studies involving human participants were reviewed and approved by Institutional Review Board of Peking University Third Hospital. Written informed consent for participation was not required for this study in accordance with the national legislation and the institutional requirements.

## Author Contributions

XL and CW supervised the project. JY, HX, and YL developed the original idea and wrote the manuscript. HX and YL conducted the statistical analysis. XW, HY, QH, YG, YD, and YW collected the data. All authors contributed to the article and approved the submitted version.

## Funding

This study was supported by Natural Science Foundation of Beijing, China (Grant Number: 7202229).

## Conflict of Interest

The authors declare that the research was conducted in the absence of any commercial or financial relationships that could be construed as a potential conflict of interest.

## Publisher's Note

All claims expressed in this article are solely those of the authors and do not necessarily represent those of their affiliated organizations, or those of the publisher, the editors and the reviewers. Any product that may be evaluated in this article, or claim that may be made by its manufacturer, is not guaranteed or endorsed by the publisher.
