# Supplementary material for: Ellipsoid Zone and External Limiting Membrane-Related Parameters on Spectral Domain-Optical Coherence Tomography and Their Relationships With Visual Prognosis After Successful Macular Hole Surgery
Source: Front Med (Lausanne). 2021 Nov 10;8:779602. doi: 10.3389/fmed.2021.779602 (PMC8631427; doi:10.3389/fmed.2021.779602)
Supplement: Supplementary file 1 [file Data_Sheet_1.docx]

**SUPPLEMENTARY MATERIAL**

Supplementary Table 1. Changes of postoperative parameters in Group A and Group B.

|  | Duration | Group A | Group B | P value |
| --- | --- | --- | --- | --- |
| BCVA, Log MAR | 1W | 0.95±0.55 | 1.27±0.78 | 0.186 |
|  | 1M | 0.75±0.24 | 1.03±0.25 | 0.001** |
|  | 6M | 0.45±0.24 | 1.00±0.20 | <0.001*** |
| Diameter of ELM disruption | 1W | 808.46±518.75 | 1198.74±713.56 | 0.090 |
|  | 1M | 564.09±684.45 | 916.55±583.24 | 0.095 |
|  | 6M | 223.96±532.62 | 517.62±573.89 | 0.096 |
| Diameter of EZ disruption | 1W | 1226.73±633.66 | 1518.82±776.26 | 0.269 |
|  | 1M | 802.92±955.60 | 1174.13±679.85 | 0.187 |
|  | 6M | 326.03±535.57 | 685.71±583.26 | 0.045* |
| ELM thickness | 1W | 0.70±3.29 | 0.00±0.00 | 0.509 |
|  | 1M | 6.05±7.69 | 1.12±4.35 | 0.009** |
|  | 6M | 11.46±6.82 | 4.24±6.42 | 0.001** |
| EZ thickness | 1W | 0.00±0.00 | 0.00±0.00 | - |
|  | 1M | 6.41±10.15 | 0.00±0.00 | 0.002** |
|  | 6M | 13.75±10.75 | 1.35±5.25 | <0.001*** |
| Fovea ILM-RPE thickness | 1W | 203.82±65.62 | 210.05±44.85 | 0.788 |
|  | 1M | 215.05±66.66 | 198.57±58.85 | 0.422 |
|  | 6M | 222.06±58.53 | 190.83±59.93 | 0.101 |
| Absolute RPE reflectivity | 1W | 214.85±21.21 | 212.63±27.21 | 0.803 |
|  | 1M | 203.81±19.00 | 207.63±28.11 | 0.593 |
|  | 6M | 207.07±21.49 | 195.77±20.05 | 0.096 |
| Absolute ELM reflectivity | 1W | 60.72±26.37 | 59.28±37.28 | 0.901 |
|  | 1M | 74.26±26.18 | 61.96±25.01 | 0.139 |
|  | 6M | 89.47±30.37 | 68.07±30.81 | 0.012* |
| Absolute EZ reflectivity | 1W | 64.60±27.93 | 61.28±30.48 | 0.764 |
|  | 1M | 88.96±52.79 | 60.23±29.40 | 0.024* |
|  | 6M | 116.68±49.07 | 62.50±36.27 | <0.001*** |
| Relative ELM reflectivity | 1W | 0.28±0.11 | 0.27±0.17 | 0.857 |
|  | 1M | 0.36±0.13 | 0.30±0.13 | 0.118 |
|  | 6M | 0.43±0.12 | 0.32±0.14 | 0.015* |
| Relative EZ reflectivity | 1W | 0.30±0.12 | 0.28±0.13 | 0.728 |
|  | 1M | 0.44±0.26 | 0.29±0.13 | 0.015* |
|  | 6M | 0.56±0.22 | 0.31±0.16 | <0.001*** |


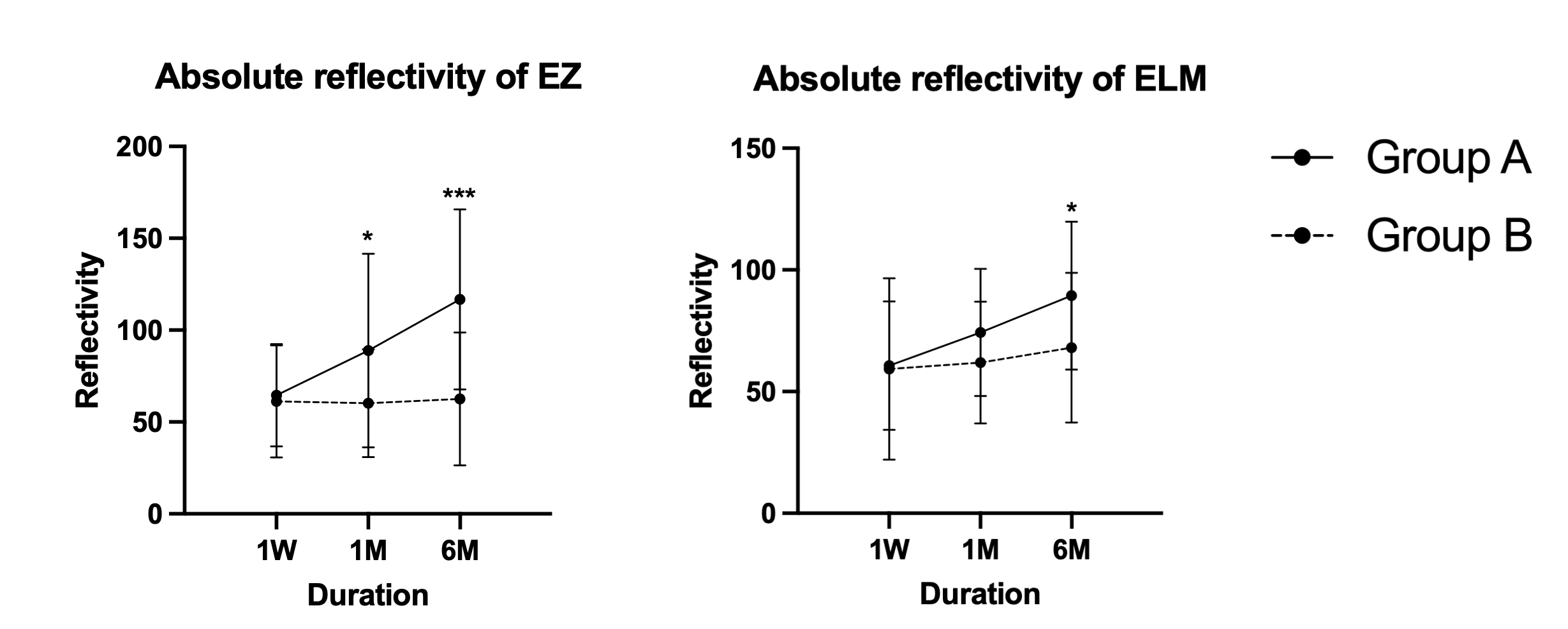


Supplementary Figure 1. Absolute reflectivity of EZ and ELM.
